# Evolution favours aging in populations with assortative mating and in sexually dimorphic populations

**DOI:** 10.1038/s41598-018-34391-x

**Published:** 2018-10-30

**Authors:** Peter Lenart, Julie Bienertová-Vašků, Luděk Berec

**Affiliations:** 1 Department of Pathological Physiology, Faculty of Medicine, Masaryk University, Kamenice 5, Building A18, 625 00 Brno, Czech Republic; 2 Research Centre for Toxic Compounds in the Environment, Faculty of Science, Masaryk University, Kamenice 5, Building A29, 625 00 Brno, Czech Republic; 3 Centre for Mathematical Biology, Institute of Mathematics, Faculty of Science, University of South Bohemia, Branišovská 1760, 37005 České Budějovice, Czech Republic; 4 Czech Academy of Sciences, Biology Centre, Institute of Entomology, Department of Ecology, Branišovská 31, 37005 České Budějovice, Czech Republic

## Abstract

Since aging seems omnipresent, many authors regard it as an inevitable consequence of the laws of physics. However, recent research has conclusively shown that some organisms do not age, or at least do not age on a scale comparable with other aging organisms. This begets the question why aging evolved in some organisms yet not in others. Here we present a simulation model of competition between aging and non-aging individuals in a sexually reproducing population. We find that the aging individuals may outcompete the non-aging ones if they have a sufficiently but not excessively higher initial fecundity or if individuals mate assortatively with respect to their own phenotype. Furthermore, the aging phenotype outcompetes the non-aging one or resists dominance of the latter for a longer period in populations composed of genuine males and females compared to populations of simultaneous hermaphrodites. Finally, whereas sterilizing parasites promote non-aging, the effect of mortality-enhancing parasites is to enable longer persistence of the aging phenotype relative to when parasites are absent. Since the aging individuals replace the non-aging ones in diverse scenarios commonly found in nature, our study provides important insights into why aging has evolved in most, but not all organisms.

## Introduction

Biological aging is defined as the age-dependent increase in the risk of death^1^. And while aging is certainly a widespread process, we now know that it is far from universal^2–6^. This is extremely interesting from the perspective of evolutionary biology, since it shows that evolution may produce non-aging or at least extremely slowly aging species. A proper understanding of why most species have evolved to age while some species have instead evolved to stay forever young may also help reveal how the aging process works and even how it can be modulated.

The first inquiries into the evolution of aging took place in the late 19th century^7^. However, only in 1951 did Peter Medawar postulate that the force of natural selection declines with age, thus providing a key impulse which led to the formation of currently mainstream “classical” evolutionary theories of aging^8^. Subsequently, Medawar’s ideas were expanded by Williams who in 1957 proposed the hypothesis of antagonistic pleiotropy, stating that evolution will select genes which are beneficial in early life even if they are deleterious later on^9^. The second key expansion of Medawar’s ideas, known as the disposable soma theory, was formulated in the 1970s^10,11^. The disposable soma theory assumes that due to a finite amount of resources, there is a trade-off between investment in reproduction and somatic maintenance. Since the optimal investment in maintenance is always found not to repair all of the somatic damage, a time-dependent deterioration, i.e. aging, occurs^12^.

Classical evolutionary theories of aging consider aging to be an inevitable by-product of evolution. Therefore, while logically consistent, they are unable to explain the existence of non-aging species. For this and other reasons, several authors have proposed that aging is directed by an aging program^13–20^. However, since the classical view of the evolution of aging precludes the evolution of such a program, alternative views of how aging might be favoured by natural selection have also been suggested^15,18,21^. For example, Josh Mitteldorf proposed that aging has evolved in order to stabilize population dynamics^14,21,22^. Furthermore, several mathematical models have demonstrated that, under specific conditions, aging can be selected by evolution^23–26^. However, these models have a limited applicability. Some assume that even without aging fertility decreases in an age-dependent manner^23^ or that competitive fitness declines with age^25^ and thus in a certain sense require aging for the evolution of aging. More importantly, the methodology, assumptions, and conclusions of each of these models have been called into question and challenged in some detail^27^. Last but not least, though the results of these modelling studies are interesting, they all simulate the evolution of aging in asexually reproducing organisms; their results thus have limited applicability for the evolution of aging in sexually reproducing organisms.

We have previously proposed a verbal model which states that although aging is, in essence, inevitable and results from damage accumulation rather than from a specific program, the actual rate of aging in nature may still be adaptive to some extent^28^. Our verbal model also briefly described how aging individuals might outcompete seemingly non-aging individuals under strong parasite pressure or rapidly changing environmental conditions. In this article, we develop a quantitative mathematical model which simulates competition between aging and non-aging individuals in a sexually reproducing population formed either by simultaneous hermaphrodites or by males and females under several ecological scenarios. We show that while advantage of aging is not obvious, several mechanisms favour aging over non-aging or at least make the aging phenotype a tougher competitor of the non-aging one.

## Methods

In this study, we independently examine effects of three ecological processes on the competition between aging and non-aging individuals: (i) absolute and relative fecundity of the aging vs. non-aging individuals, (ii) individuals’ mating preferences for the same or opposite phenotype, and (iii) various impacts of parasitism. In addition, we run the corresponding simulation scenarios under two types of sexual reproduction, in populations formed by simultaneous hermaphrodites or by genuine males and females.

**Figure 1:**
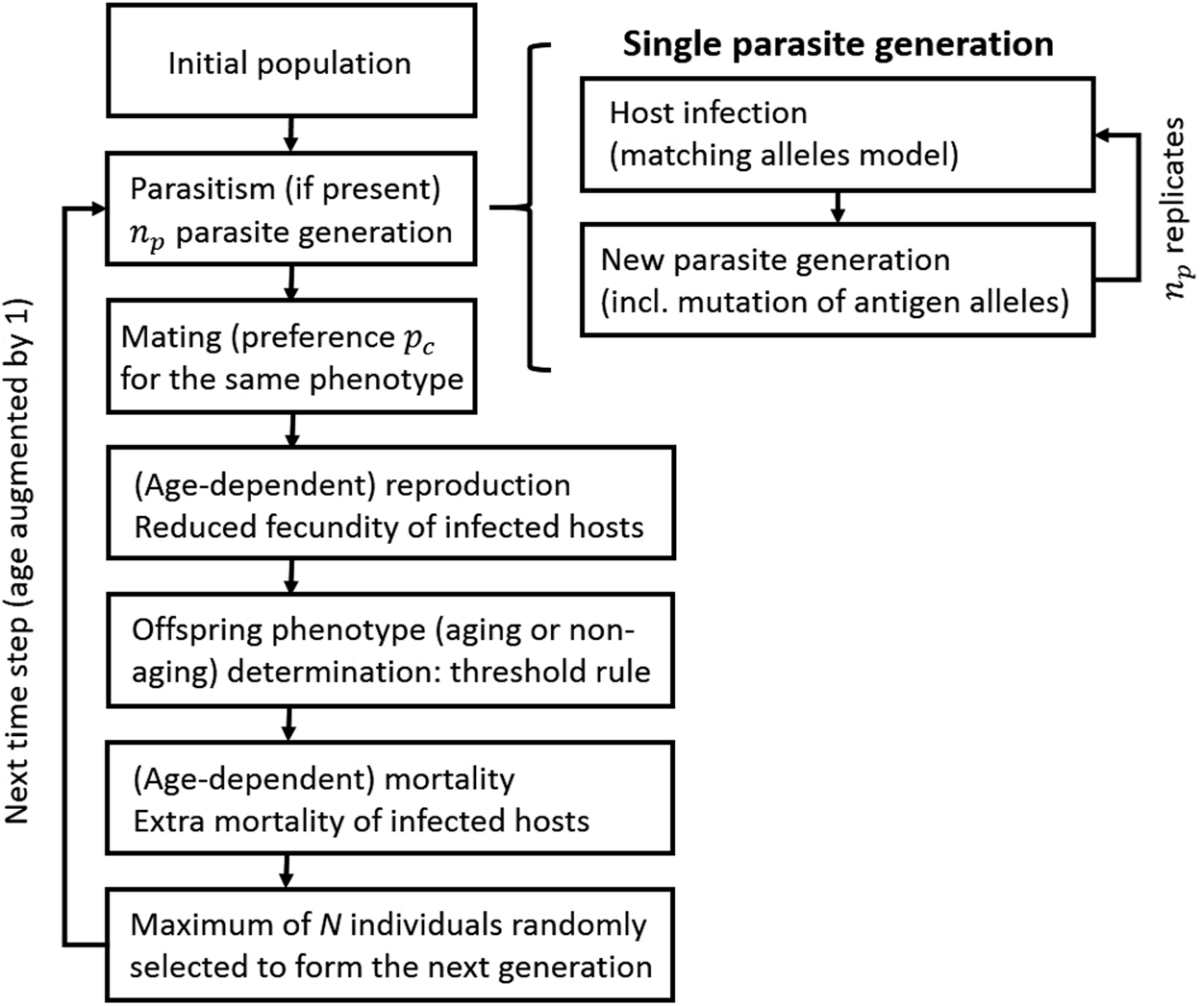
Flowchart of our simulation model. For details see the Methods section.

We begin with a population composed of *N* simultaneous hermaphrodites of two phenotypes: aging and non-aging. Our model is an agent-based simulation model that allows phenotypes of all individuals to be modelled explicitly and their competitive dynamics to be followed over time. Time is discrete, with the time step corresponding to the age increment of 1 and with all relevant rates and probabilities defined on a per time step basis. Simulations are run for *T* time steps and each scenario is replicated a given number of times. All model parameters and variables are summarized in Table 1. Moreover, a model flowchart is provided in Fig. 1 and explained in detail below in this section. The model is then modified to include populations composed of males and females. We note that our models are modifications of the classical models of the evolution of sexual reproduction^29–32^ which moreover take into account individual age and aging and non-aging phenotypes.

**Table 1:** Default parameter and variable settings used in the hermaphrodite model.

Parameter or variable	Meaning	Value(s)
*N*	Fixed (host) population size	1000
*T*	Simulation time	500/1000
*a*	Individual age	varies
*d*(*a*)	Per time step probability of dying at age *a* for aging individuals	Eqn 1
*d*_0_	Per time step probability of dying at age 1 for aging individuals	0.01
*k*_*d*_	Rate at which mortality of aging individuals increases with age	10
*b*(*a*)	Fecundity of aging individuals at age *a*	Eqn 2
*b*_0_	Fecundity of aging individuals at age 1	1.5/3/6
*k*_b_	Rate at which fecundity of aging individuals decreases with age	0.5
*δ*	Age-independent per time step probability of dying of non-aging individuals	*d*_0_ ( = 0.01)
*β*	Age-independent fecundity of non-aging individuals	varies
*n*_g_	Number of phenotype loci	10
*n*_*i*_	Number of immunity loci for hosts and antigen loci for parasites	1
*n*_*a*_	Number of different immunity/antigen alleles	4
*p*_*f*_	Specific proportion of phenotype loci that have to contain the allele 1 for the individual to be non-aging	0.8
*p*_*g*_	Minimum proportion of alleles in the phenotype genome that are of type 1 for the individual to be non-aging	0.9
*P*	Fixed parasite population size	2000
*μ*_*a*_	Per haplotype locus mutation probability for the antigen genome of the parasite	0.03
*E*_1_	Fecundity reduction of infected individuals	0.8/0
*E*_2_	Additional per time step mortality probability of infected hosts	0/0.2/0.4
*n*_*p*_	Number of non-overlapping parasite generations per time step	2
*p*_*s*_	Starting proportion of aging individuals	0.5
*p*_*c*_	Preference of aging individuals for aging mates and of non-aging individuals for non-aging mates	0.5

### Aging and non-aging phenotypes

Aging and non-aging phenotypes differ according to demographic rates. Individuals are characterized by age *a*. We define the probability that an aging individual of age *a* dies during a time step as

1 $$d(a)={d}_{0}+(1-{d}_{0})\frac{(a-1)}{(a-1)+{k}_{d}}.$$

This function increases from 0 < *d*_0_ < 1 for *a* = 1 to 1 as *a* grows large. Similarly, we define the fecundity of an aging individual as

2 $$b(a)=\frac{{b}_{0}}{1+{k}_{b}(a-1)}.$$

Hence, the fecundity *b*(*a*) declines with age from *b*_0_ > 0 for *a* = 1 to 0 as *a* grows large. Mortality of newborns in their first year of life is included in the parameter *b*_0_ and equations (1) and (2) are thus applicable to individuals of age *a* > 0. Non-aging individuals die during a time step with an age-independent probability *δ* and produces *β* offspring in each time step, irrespective of age.

### Genetics

In addition to age, each individual is characterized by two genome portions. The phenotype genome, composed of *n*_*g*_ loci, serves to determine whether a newborn individual is of the aging or non-aging phenotype (see below). The role of the immunity genome, composed of *n*_*i*_ loci, is to check for an individual’s resistance to a parasitic infection (see below). Both genome portions are assumed haploid. Also, individuals are characterized as susceptible or infected (all are initially susceptible, and all are born susceptible).

Any phenotype genome locus may feature one of two allele types, with 0 and 1 denoting alleles contributing to the aging and non-aging phenotype, respectively. There are many viable options how to determine the aging and non-aging phenotype from a genome. However, it is important to realize that there are several interconnected causes of aging^33,34^ including DNA double-strand breaks (DSBs)^35,36^, Telomere attrition^37^, decreased proteasomal activity^38^ and others^39^. Therefore, any non-aging organism has to be equipped with several fine-tuned molecular pathways repairing damage from both intrinsic and extrinsic sources. Furthermore, it is likely that disturbance of any of these pathways would result in an aging phenotype. In accordance with this line of reasoning, we assume in our model that a number of phenotype loci must be in harmony to produce a non-aging phenotype; as a result, we have opted for adopting a threshold rule. In particular, two conditions must be met for an individual to be considered non-aging: (i) the specific proportion *p*_*f*_ of phenotype loci (e.g. the first *p*_*f*_*n*_*g*_ loci) must contain the allele 1, and (ii) the proportion of all phenotype loci that harbour the type 1 allele must exceed a given threshold value *p*_*g*_ > *p*_*f*_.

In the immunity genome, alleles denoted as 0, 1, …, *n*_*a*_ − 1 represent *n*_*a*_ alternative variants of the immunity allele. The phenotype and immunity genomes of each offspring are determined following the free recombination of their parents’ genomes and a random choice of one of two resulting genomes. No mutations are assumed to occur on the phenotype and immunity genomes. For the phenotype genome, alleles at all loci of aging individuals are initially of type 0 and alleles at all loci of non-aging individuals are initially of type 1. Individuals are initialized with an allele randomly selected for each locus in the immunity genome.

### Parasites

For scenarios examining effects of parasites on the competition between aging and non-aging phenotypes, we consider a parasite population of a fixed size *P*. Individual parasites are characterized by the haploid antigen genome, with *n*_*a*_ alleles denoted as 0, 1, …, *n*_*a*_ − 1 that are initially randomly distributed across the *n*_*i*_ loci. Genetic variation at the antigen loci is maintained by setting the per locus mutation rate to *μ*_*a*_ per parasite generation. When a mutation occurs at a locus, the existing allele is replaced by an allele randomly chosen from the *n*_*a*_ alleles 0, 1, …, *n*_*a*_ − 1. Parasites affect their hosts either by reducing their fecundity by a factor *E*_1_ or increasing their mortality via imposing an extra probability *E*_2_ of dying during the time step.

Since parasite life cycles are commonly faster than those of their hosts, we assume there are *n*_*p*_ non-overlapping parasite generations per time step. During each parasite generation, hosts are drawn sequentially in a random order and each is exposed to a randomly selected parasite. We use a matching alleles model of infection genetics^29–32,40,41^ to establish whether the exposed host is actually infected: if the immunity genome of the host and the antigen genome of the parasite match exactly at all loci, the parasite establishes infection in the host, otherwise it dies. Hosts in which infection is established are marked as infected. At the end of each parasite generation, *P* new parasites appear in the environment by drawing individuals at random (with replacement) from among the successful parasites. Following introduction of a new parasite, its antigen alleles may mutate to any of the *n*_*a*_ alleles 0, 1, …, *n*_*a*_ − 1, each with the probability *μ*_*a*_.

### Host demography

The parasitic phase is followed by host demography. First, individuals mate and reproduce. There is a preference *p*_*c*_ of aging individuals for aging mates and of non-aging individuals for non-aging mates, but otherwise mates are chosen randomly. We note that *p*_*c*_ = 0.5 indicates no mating preference and thus a random choice of mates by all host individuals regardless of phenotype. Upon mating, a Poisson-distributed number of offspring are produced, with mean *b*(*a*) and *β* for the aging and non-aging individuals, respectively. This number is reduced by the factor 1 − *E*_1_ if the reproducing host is infected. The offspring are born susceptible, with age 1 (as we emphasize earlier, fecundity already accounts for the first-year mortality), and the phenotype genome of each offspring are determined (see above). The phenotype (aging or non-aging) of each offspring is then determined using the above-described threshold rule.

Background mortality of other than the newborn hosts then occurs: the aging and non-aging individuals die with probability *d*(*a*) and *δ*, respectively. This is followed by the extra mortality probability *E*_2_ of the infected hosts. The age of all surviving individuals is augmented by 1 and we record the numbers of aging and non-aging individuals. Eventually, a maximum of *N* individuals is randomly selected to form the population appearing at the beginning of the next time step.

### Two-sex model version

To adapt our model for males and females, some of its components are replaced and a few new elements are introduced. Aside from these modifications, the principles of the two-sex model mirror those of the above hermaphrodite model. First, we replace hermaphrodites with females and males. Therefore, females select their mating partners from among males and only females produce offspring. Since simultaneous hermaphrodites need to allocate some amount of resources to both male and female functions, then – relative to females – their fecundity is commonly assumed to be reduced, as much as by one half^42^. Therefore, to fairly compare simulations of the two model versions, we assume female fecundity to be twice that of hermaphrodites. Both females and males are assumed to have identical mortality patterns, depending on whether they are aging or non-aging.

The phenotype genome in the sexually dimorphic population is diploid, with the non-aging alleles assumed to be relatively recessive. Again, we adopt a threshold rule where two conditions must be met for an individual to be non-aging: (i) the specific proportion *p*_*f*_ of phenotype loci must be homozygous for the allele 1, and (ii) the proportion of alleles 1 in the entire phenotype genome must exceed a given threshold value *p*_*g*_ > *p*_*f*_.

## Results

### Effects of fecundity

Both absolute and relative values of fecundity were found to determine the outcome of competition between aging and non-aging individuals of a simultaneous hermaphrodite. Assuming no parasites (*P* = 0) and no mating preferences (*p*_*c*_ = 0.5), and setting the (initial) fecundity (and mortality) of aging and non-aging individuals to the same values, non-aging individuals always won the competition and expelled the aging phenotype out of the population (Supplementary Fig. S1). Still, the aging individuals remained in the population the longer the higher was their initial frequency in the population (Supplementary Fig. S1). We note here that an initial increase in the number of aging individuals in the first few generations, observed in Supplementary Fig. S1 as well as in nearly all figures that follow, is a consequence of our threshold approach to modeling inheritance of aging and of our assumption that at the start of every simulation, the phenotype loci of all aging and non-aging individuals contain solely aging or non-aging alleles, respectively.

**Figure 2:**
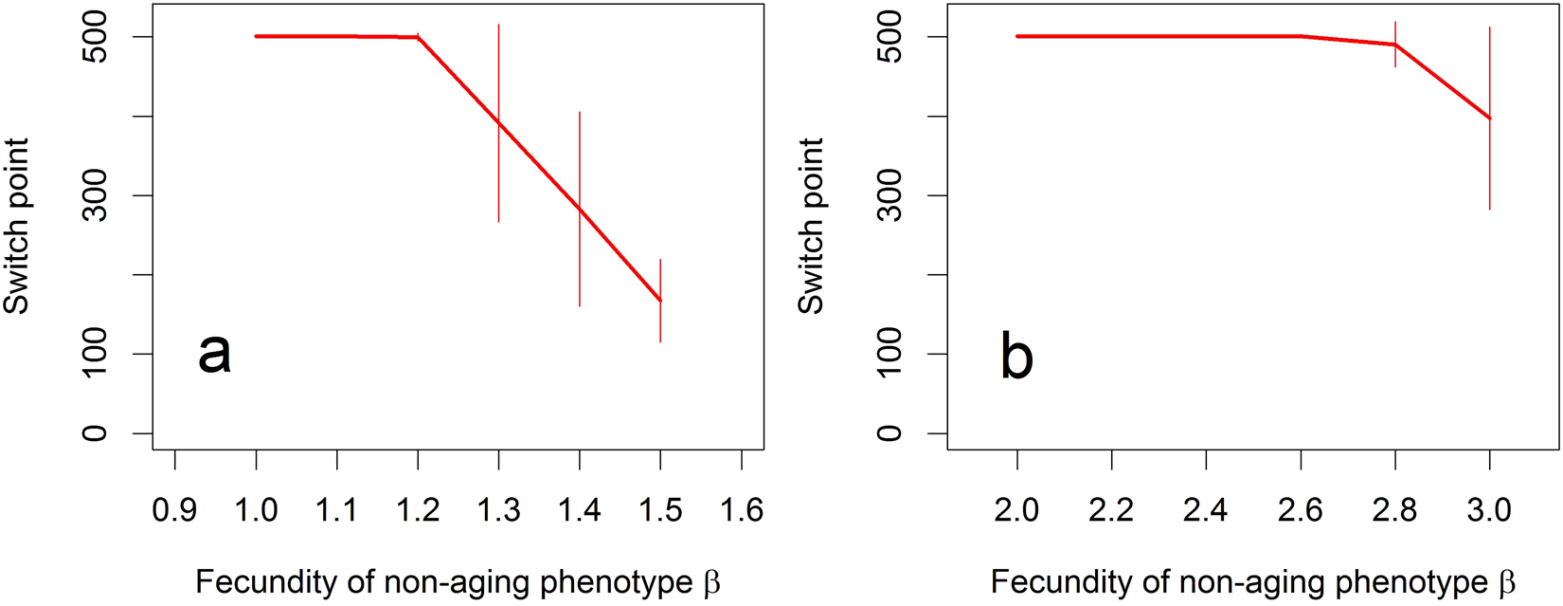
Mean and one standard deviation of the time instant, termed the switch point here, at which the non-aging phenotype becomes more abundant than the aging one (and eventually expels the latter). Switch point equal to 500 means that this did not happen before the end of simulation run, which nonetheless nearly always corresponded to the 100% dominance of the aging phenotype. Parameter values: (**a**) *b*_0_ = 1.5 and (**b**) *b*_0_ = 3; other parameters are as in Table 1, with *p*_*s*_ = 0.5, *p*_*c*_ = 0.5, and *P* = 0. Based on 20 simulation runs for each simulated scenario.

The aging phenotype may win the competition if its initial fecundity is sufficiently high relative to that of the non-aging one. For example, setting the initial fecundity *b*_0_ of aging individuals to 1.5, we find that as the fecundity *β* of non-aging phenotype declines, it takes longer for the latter to expel the aging phenotype, until the 100% domination of the aging phenotype is eventually observed after *T* = 500 generations for *β* = 1.1(Fig. 2a). Thus, the minimum difference 0.4 in the (initial) fecundity which enabled domination of the aging phenotype in this specific setting was about 27% of the initial fecundity of the aging phenotype. However, for the initial fecundity b_0_ of aging individuals set to 3 the aging phenotype won the competition already when *β* = 2.6, corresponding to only about 13% reduction in the fecundity of non-aging phenotype relative to the initial fecundity of the aging one (Fig. 2b).

We use identical values of *b*_0_ and *β* further on for aging and non-aging phenotypes, respectively, to reveal mechanisms that may be to the advantage of the aging phenotype and are not confounded by any demographic differences between the aging and non-aging individuals than aging itself. For the same reason, we also use *p*_*s*_ = 0.5, meaning that initially there is an equal number of aging and non-aging individuals in the population.

### Effects of mating preferences

**Figure 3:**
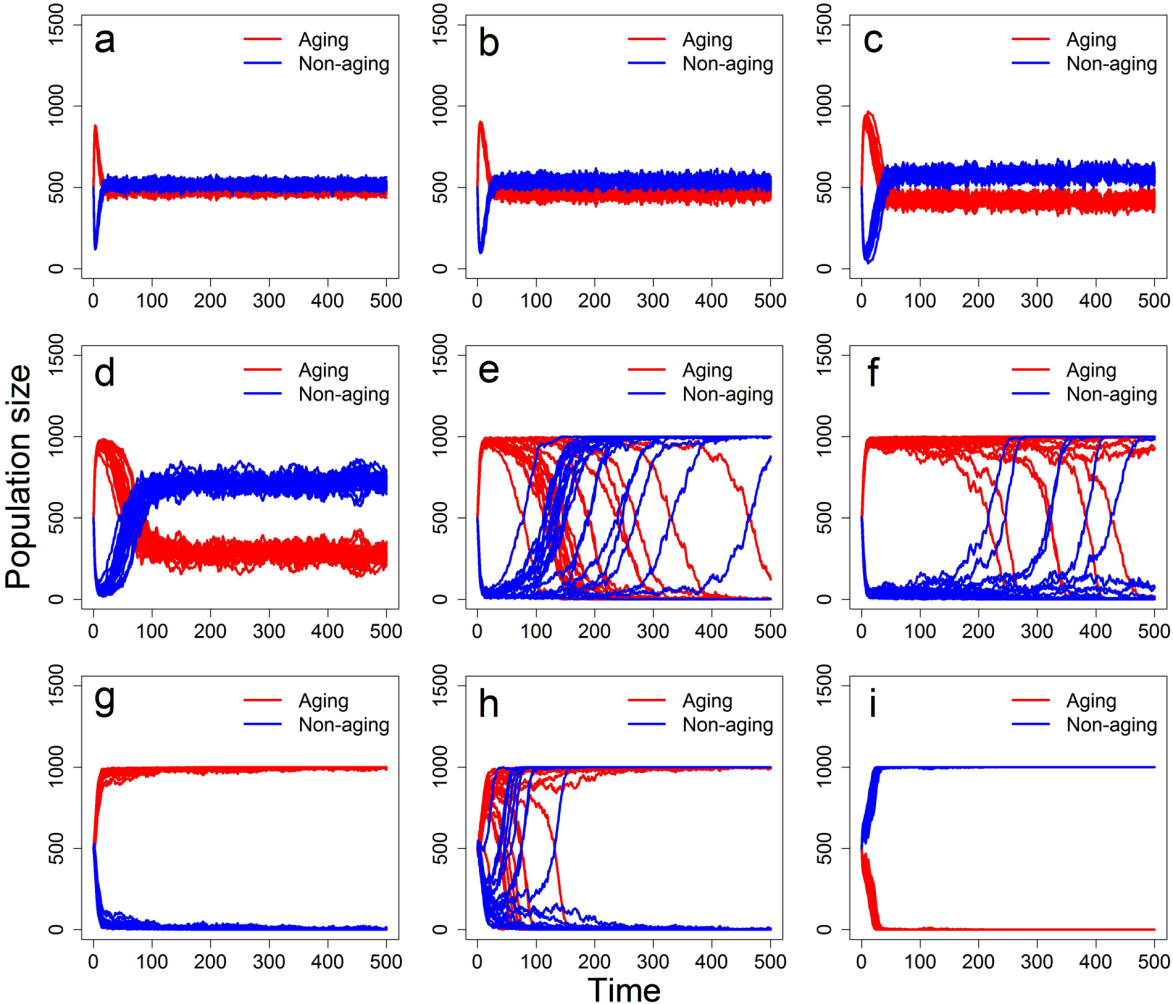
Representation of the aging and non-aging phenotypes in the population composed of simultaneous hermaphrodites under a variety of mating preferences. Results are based on 20 simulation replicates and mating preferences are equal to (**a**) *p*_*c*_ = 0.1, (**b**) *p*_*c*_ = 0.2, (**c**) *p*_*c*_ = 0.3, (**d**) *p*_*c*_ = 0.4, (**e**) *p*_*c*_ = 0.5, (**f**) *p*_*c*_ = 0.6, (**g**) *p*_*c*_ = 0.7, (**h**) *p*_*c*_ = 0.8, and (**i**) *p*_*c*_ = 0.9. Other parameters are as in Table 1, with *b*_0_ = *β* = 1.5, *p*_*s*_ = 0.5, and *P* = 0.

Here we test the effect of mating preferences by varying the parameter *p*_*c*_, assuming equal (initial) fecundities of aging and non-aging phenotypes (*b*_0_ = *β*) and no parasites (*P* = 0). We recall that when no mating preferences exist in the population for (non-)aging phenotypes (*p*_*c*_ = 0.5), the non-aging individuals always win the competition and expel the aging phenotype from the population. To further elucidate meaning of the parameter *p*_*c*_, e.g. *p*_*c*_ = 0.3 corresponds to the 70% probability of mating with the opposing phenotype (aging with non-aging and vice versa; disassortative mating) while *p*_*c*_ = 0.7 corresponds to the 70% probability of mating with the same phenotype (assortative mating). Generally, mating preferences for the opposing phenotype (*p*_*c*_ > 0.5) give rise to coexistence equilibrium in the proportion of aging and non-aging phenotypes (Fig. 3). On the other hand, for a range of *p*_*c*_ values corresponding to assortative mating (*p*_*c*_ > 0.5) the aging phenotype is favoured (Fig. 3). Thus, unless the mating preferences for the same phenotype are high, we observe the rapid domination of the aging phenotype. Finally, strong enough assortative mating (high enough values of *p*_*c*_) results in a fast and stable domination of the non-aging phenotype (Fig. 3).

**Figure 4:**
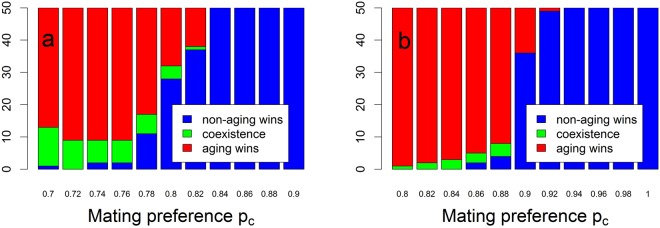
The outcome of competition between aging and non-aging phenotypes in the hermaphrodite population on a finer scale of the mating preference parameter *p*_*c*_ and the location of its value at which the outcome tips from aging phenotype to non-aging phenotype domination. Other parameters are as in Table 1, with (**a**) *b*_0_ = *β* = 1.5, (**b**) *b*_0_ = *β* = 3, *p*_*s*_ = 0.5, and *P* = 0; based on 50 simulation replicates for each simulated scenario.

In addition to this unexpected and non-trivial impacts of mating preferences on the outcome of competition between the aging and non-aging phenotypes, we observe an interplay between their (initial) fecundity and the strength of mating preferences on at what value of *p*_*c*_ the assortative mating tips the outcome from aging phenotype to non-aging phenotype domination. Adopting a finer resolution of the parameter *p*_*c*_, we find that as the (initial) fecundity *b*_0_ = *β* increases, the tipping point of *p*_*c*_ increases, too (Fig. 4).

Qualitatively, the effect of mating preferences stays unchanged unless the two parameters determining the threshold rule, *p*_*f*_ and *p*_*g*_, are relatively low (Supplementary Fig. 2). We note that a decrease in these parameters corresponds to a less strict rule of determining the offspring phenotype. Relatively low values of these parameters are thus in contrast to our assumption that non-aging requires a sort of fine-tuning of several molecular mechanisms (we provide some support of this assumption in the Methods section).

### Effects of parasitism

**Figure 5:**
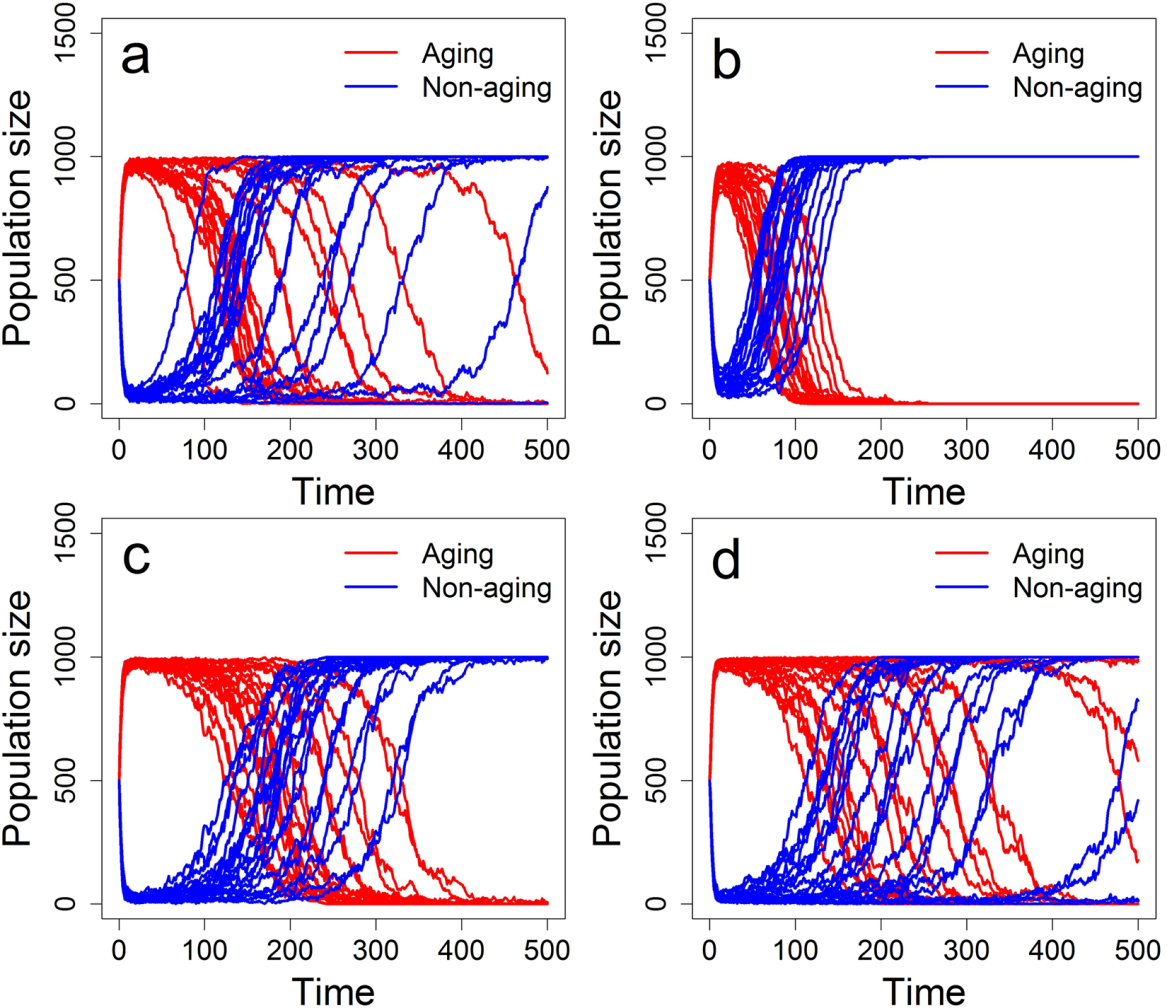
Representation of the aging and non-aging phenotypes in the hermaphrodite population under various parasite pressure. Results show a scenario without parasites (**a**), with parasites causing a fecundity reduction (**b**; *E*_1_ = 0.8, *E*_2_ = 0), and with parasites causing mortality enhancement (**c**; *E*_1_ = 0, *E*_2_ = 0.2, and **d**; *E*_1_ = 0, *E*_2_ = 0.4). Other parameters are as in Table 1, with *b*_0_ = *β* = 1.5, *p*_*c*_ = 0.5, *p*_*s*_ = 0.5; 20 simulation replicates are run for each simulated scenario.

To test whether a parasite pressure affects competition of the aging and non-aging phenotypes we have tested several scenarios. In the first scenario, there was no parasite present. In the second scenario, the parasite caused 80% fecundity reduction in the infected hosts, while in the third and fourth scenarios the infection by parasite led to 20% or 40% percent extra chance of death, respectively. Relative to the case of no parasites (Fig. 5a), a parasite-induced decline in fecundity accelerates domination of the non-aging phenotype (Fig. 5b) while a parasite-induced increase in mortality has the opposite effect (Fig. 5c,d).

### Effects of mating system

**Figure 6:**
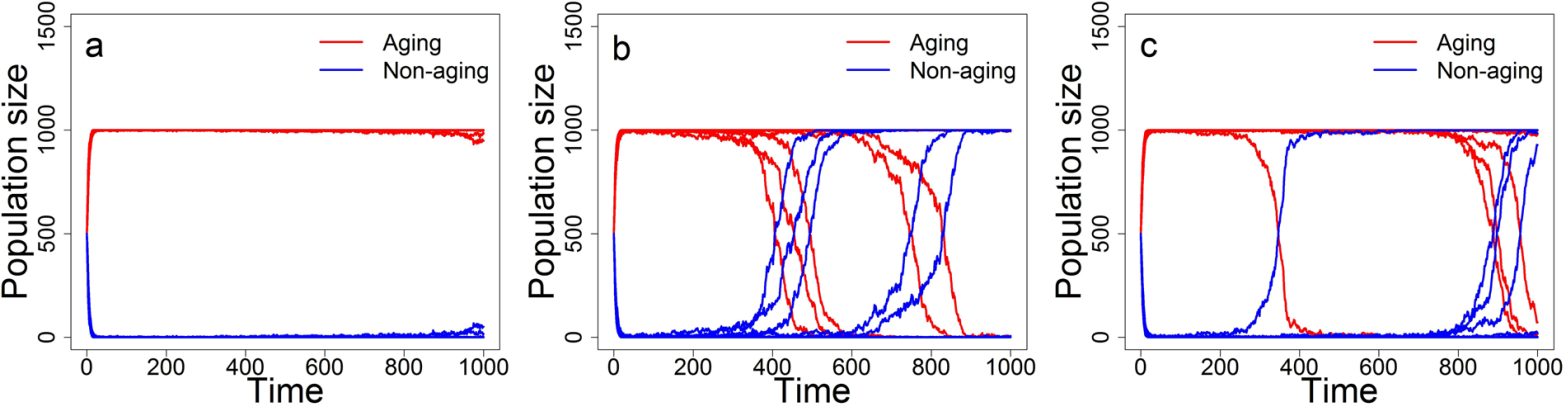
Representation of the aging and non-aging phenotypes in the population composed of males and females, for different fecundities of the non-aging phenotype: (**a**) *β* = 2 × 1.3, (**b**) *β* = 2 × 1.4, (**c**) *β* = 2 × 1.5. The initial fecundity of the aging phenotype is here set to *b*_0_ = 2 × 1.5. Other parameters are as in Table 1, with *p*_*s*_ = 0.5, *p*_*c*_ = 0.5, and *P* = 0; 20 simulation replicates are run for each simulated scenario.

Haploid simultaneous hermaphrodite is a typical model organism used in theoretical evolutionary studies. Here we test whether and how our previous simulation results change when we instead consider the two-sex model version with the population composed of diploid males and females. First, we test effects of different values of (initial) fecundity of aging and non-aging individuals. Also here, just a relatively small difference in the (initial) fecundity in favour of the aging phenotype was observed to lead to its long-term domination (Fig. 6). However, if the non-aging phenotype was to win the competition in the two-sex population (even under no difference in (initial) fecundity) time required for it to dominate was much longer than in comparable simulations with simultaneous hermaphrodites. Indeed, even after 1000 generations, the aging phenotype was more frequent in most simulations (Fig. 6). Sexual dimorphism is thus vastly more favourable for the aging phenotype than simultaneous hermaphroditism.

**Figure 7:**
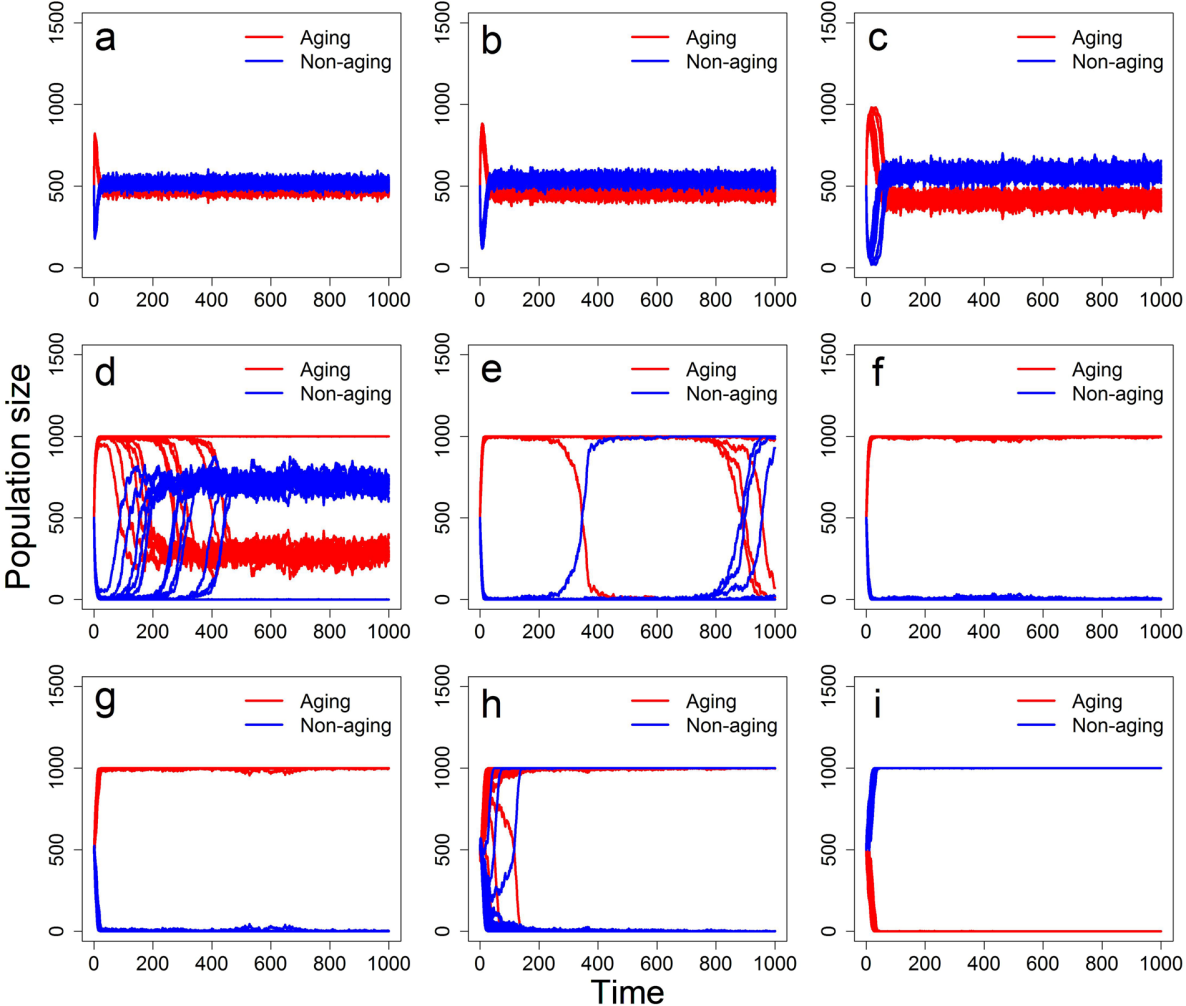
Representation of the aging and non-aging phenotypes in the two-sex population under a variety of mating preferences. Results are based on 20 simulation replicates and mating preferences are equal to (**a**) *p*_*c*_ = 0.1, (**b**) *p*_*c*_ = 0.2, (**c**) *p*_*c*_ = 0.3, (**d**) *p*_*c*_ = 0.4, (**e**) *p*_*c*_ = 0.5, (**f**) *p*_*c*_ = 0.6, (**g**) *p*_*c*_ = 0.7, (**h**) *p*_*c*_ = 0.8, and (**i**) *p*_*c*_ = 0.9. Other parameters are as in Table 1, with *b*_0_ = *β* = 3, *p*_*s*_ = 0.5, and *P* = 0.

Next, we test effects of mating preferences. As in the case of simultaneous hermaphrodites, mating preferences for the opposite phenotype (*p*_*c*_ < 0.5) led to the formation of coexistence equilibrium in the frequency of aging and non-aging phenotypes, while not strong enough assortative mating (*p*_*c*_ > 0.5) led to permanent domination of the aging phenotype (Fig. 7). Finally, large values of *p*_*c*_ resulted in the fast and stable domination of the non-aging phenotype (Fig. 7). Even though we observe here the same trends as in the hermaphrodite model, also in these scenarios sexual dimorphism is much more favourable for the aging phenotype than simultaneous hermaphroditism (compare Fig. 3 and Fig. 7). The effect of sexual dimorphism on the simulation results is thus quantitative rather than qualitative. Interestingly, however, sexual dimorphism does not appear to have an influence on the location of tipping points *p*_*c*_ at which domination changes from the aging phenotype to the non-aging one (compare Fig. 4 and Supplementary Fig. S3).

Regarding effects of parasite pressure on the competition between aging and non-aging phenotypes in a two-sex population, we get results analogous to those obtained for the hermaphrodite model (compare Fig. 5 and Supplementary Fig. S4). In particular, relative to the case of no parasites, a parasite-induced decline in fecundity accelerates domination of the non-aging phenotype while a parasite-induced increase in mortality has the opposite effect and allows the aging phenotype to persist in the population for a longer period than in the case of parasite absence (Supplementary Fig. S4).

## Discussion

To the best of our knowledge, this study is the first to present a mathematical model of the evolution of aging in sexually reproducing organisms. Our results offer several insights into the evolution of aging. First, we show that a sufficiently but not excessively higher initial fecundity can outweigh fitness disadvantage associated with aging. Our second – and arguably the most interesting – result indicates that assortative mating (with regard to the phenotype) by itself leads to quick and permanent domination of the aging phenotype, whereas disassortative mating leads to the coexistence of aging and non-aging individuals. Third, whereas sterilizing parasites promote dominance of the non-aging phenotype, the effect of mortality-enhancing parasites is to enable longer persistence of the aging phenotype relative to when parasites are absent. Finally, the aging phenotype fares much better in a two-sex population than among (sexually reproducing) simultaneous hermaphrodites.

Our first core finding is that a higher (initial) fecundity of the aging individuals relative to the non-aging ones may compensate for fitness disadvantage of aging and lead to the fixation of aging phenotype. This finding can be easily integrated into the classical theories of aging. Since both the theory of antagonistic pleiotropy^9^ and the disposable soma theory^11^ predict that longevity and fecundity are in a trade-off relationship, it makes sense to assume that the aging individuals would have a higher (initial) fecundity than the non-aging ones. This result alone is therefore capable of explaining why aging is so widespread in nature. Non-aging may thus evolve in organisms where it does not significantly reduce fecundity or, in other words, where the fecundity cost to non-aging is low.

Our second core finding – which is also arguably the most surprising result of our simulations – is that assortative mating, if not too strong, leads to the fast and permanent fixation of the aging phenotype. Assortative mating constitutes a barrier in gene flow and as such may be the result of causes other than an active preference for the same phenotype. For example, if we consider a large population composed of smaller spatially divided subpopulations which are predominantly but not exclusively composed of individuals with the same phenotype, then even if all individuals choose their mating partners randomly, the mating itself is likely assortative because individuals from different subpopulations meet only occasionally. In such a case, assortative mating is the outcome of geography. Since assortative mating may be caused by many different situations, it is likely to play some role in the evolution of aging across the tree of life. On the other hand, our results also suggest that very strong assortative mating, unlike weaker assortative mating, favours the non-aging instead of the aging phenotype. While this 180-degree turn in the effect of assortative mating may seem surprising, it has a rather simple explanation. In particular, very strong assortative mating will generate two almost separate populations, which under an equal (initial) fecundity of both phenotypes leads to the rapid domination of the non-aging phenotype since it on average produces more offspring per generation than the aging one. Nevertheless, although natural populations with very strong assortative mating do exist in which almost 100% of mating occurs between similar phenotypes^43^, assortative mating is likely not nearly as strong in most species and for most phenotypes. In summary, our results suggest that in most likely and common forms assortative mating quickly results in the domination of aging phenotype. The non-aging phenotype prevails only if assortative mating becomes too strong and a polymorphism between both phenotypes arises when mating is disassortative. Thus, assortative mating on its own may be the process that may explain why most species age while some do not.

Third, our finding that parasite pressure may slow the pace at which the non-aging individuals outcompete the aging ones, is in accordance with some alternative views of the evolution of aging. For example, in our previous work, we have invoked the Red Queen hypothesis and proposed that a faster rate of aging may be selected because aging could enable faster adaptation to changing conditions, most notably coevolution with a parasite^28^. Others have also invoked the Red Queen hypothesis in a similar manner with regard to the evolution of aging^24^. Our results thus support the notion that the presence of parasites may be one of the forces driving the evolution of aging. On the other hand, our simulations modelling the competition between aging and non-aging individuals also show that parasite pressure is not strong enough to facilitate a fixation of aging phenotype. Nevertheless, competition between aging and non-aging individuals is a model situation which is sort of extreme and it is thus possible that parasite pressure may have a more profound effect on competition between individuals with different rates of aging. Also, parasite pressure can lead to the fixation of the aging phenotype if combined with other effects. Accordingly, the previously published model shows that parasite pressure can lead to the evolution of shorter lifespan in metapopulations^44^. Our results further develop this point by showing that parasite pressure may also affect the evolution of aging, and thus also the evolution of lifespan even when the population is not divided into smaller parts.

Interestingly, we find that parasites with different impact on their host may affect the course of competition differently. Indeed, whereas it is a mortality-enhancing parasite that slows the pace at which the non-aging individuals outcompete the aging ones, sterilizing parasites conversely speed up the dominance of the non-aging phenotype. One plausible interpretation for the effect of mortality-enhancing parasite is that the increase in mortality it causes reduces the main advantage of non-aging phenotype, its longer lifespan, and thus delays the domination of non-aging phenotype. On the other, the 80% fertility reduction caused by the sterilizing parasite may result in almost no offspring in the aging phenotype due to its short average lifespan and just a few offspring in the non-aging phenotype. The difference between almost no offspring and just a few offspring produced by the infected individuals may at a longer time scale be an important difference driving faster domination of the non-aging phenotype.

Our final core finding establishes that sexual dimorphism seems to be more favourable to the aging phenotype than simultaneous hermaphroditism. In accordance with this result, we make a testable prediction that a lack of senescence should be more common in hermaphroditic organisms. Otherwise, the above described results are conserved for this mating system, too. Offering a novel viewpoint on the evolution of aging, this finding also suggests that the common practice of considering haploid simultaneous hermaphrodites (or even haploid asexuals!) as a proxy for diploid females and males in population genetic models need not always be appropriate.

It is important to note that the competition between aging and non-aging individuals in our models is an extreme scenario, not only because of an existence of truly non-aging individuals but also because aging individuals in the model age relatively fast. While such situation might be unlikely to occur in nature we believe it is a useful simplification which highlights the most important effects in the evolution of aging. Indeed, it is sensible to assume that if under some circumstances the “fast aging” individuals can outcompete the non-aging ones it could be even easier for “normal aging” individuals to outcompete “slowly aging” individuals.

Overall, we propose that aging may be preferable to non-aging in several reasonably likely situations. Some of them occur at least in some populations, and others are almost universal. Since it is possible that these scenarios and effects combine in nature, aging might be preferable to non-aging for most species.

## Electronic supplementary material

Supplementary Information

## Data Availability

The datasets generated and/or analysed during the current study are available from the corresponding author on reasonable request.
