# Supplementary material for: Evolution favours aging in populations with assortative mating and in sexually dimorphic populations
Source: Sci Rep. 2018 Oct 30;8:16072. doi: 10.1038/s41598-018-34391-x (PMC6207771; doi:10.1038/s41598-018-34391-x)
Supplement: Supplementary file 1 — Supplementary Information [file 41598_2018_34391_MOESM1_ESM.pdf]

Supplementary Information for

Evolution favors aging in populations with assortative mating and in sexually dimorphic populations

Peter Lenart[1, 2], Julie Bienertová-Vašků[1, 2], Luděk Berec[3, 4*]

[1]*Department of Pathological Physiology, Faculty of Medicine, Masaryk University, Kamenice 5, Building A18, 625 00, Brno, Czech Republic*

[2]*Research Centre for Toxic Compounds in the Environment, Faculty of Science, Masaryk University, Kamenice 5, Building A29, 625 00, Brno, Czech Republic*

[3]*Centre for Mathematical Biology, Institute of Mathematics, Faculty of Science, University of South Bohemia, Branišovská 1760, 37005 České Budějovice, Czech Republic*

[4]*Czech Academy of Sciences, Biology Centre, Institute of Entomology, Department of Ecology, Branišovská 31, 37005 České Budějovice, Czech Republic*

[*]*Author for correspondence: Luděk Berec, berec@entu.cas.cz*

This supplementary file contains Supplementary Figures S1 to S4

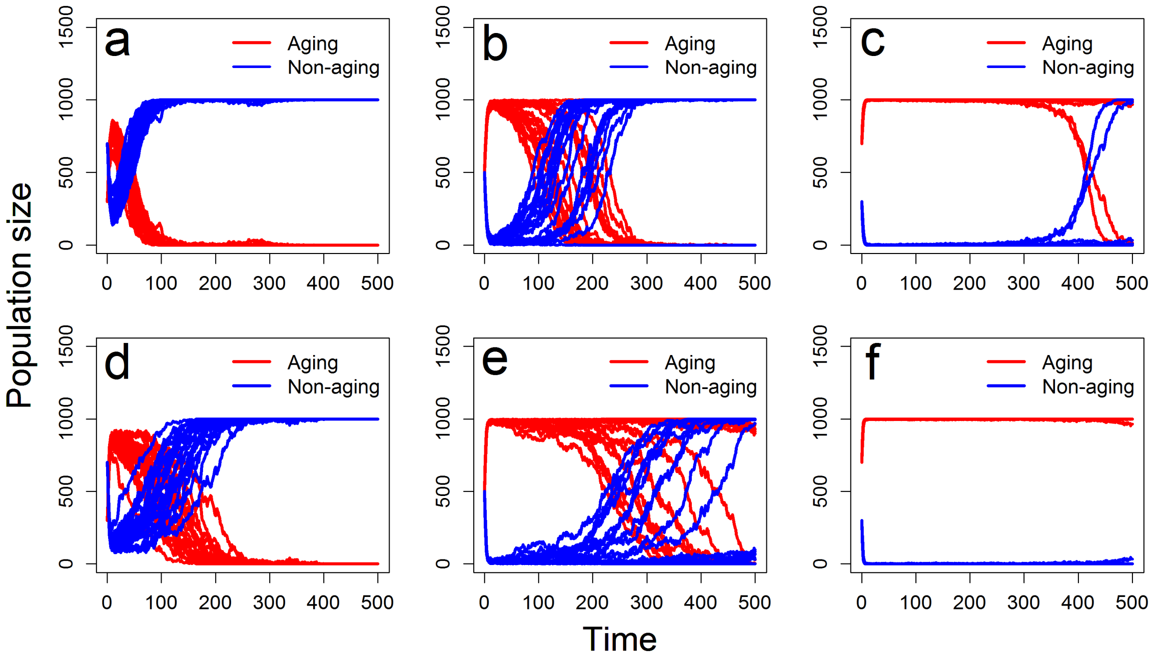

Supplementary Figure S1: Representation of aging and non-aging phenotypes in the population composed of simultaneous hermaphrodites under different (initial) fecundities and initial proportions of the aging and non-aging phenotypes. Parameter values: (a-c) $b_0 = \beta = 1.5$, (d-f) $b_0 = \beta = 3$, left column: $p_s = 0.3$, middle column: $p_s = 0.5$, right column: $p_s = 0.7$; other parameters as in Table 1 in the main text, with no mating preferences ($p_c = 0.5$) and no parasite pressure ($P = 0$); 20 simulation replicates are plotted for each simulated scenario.

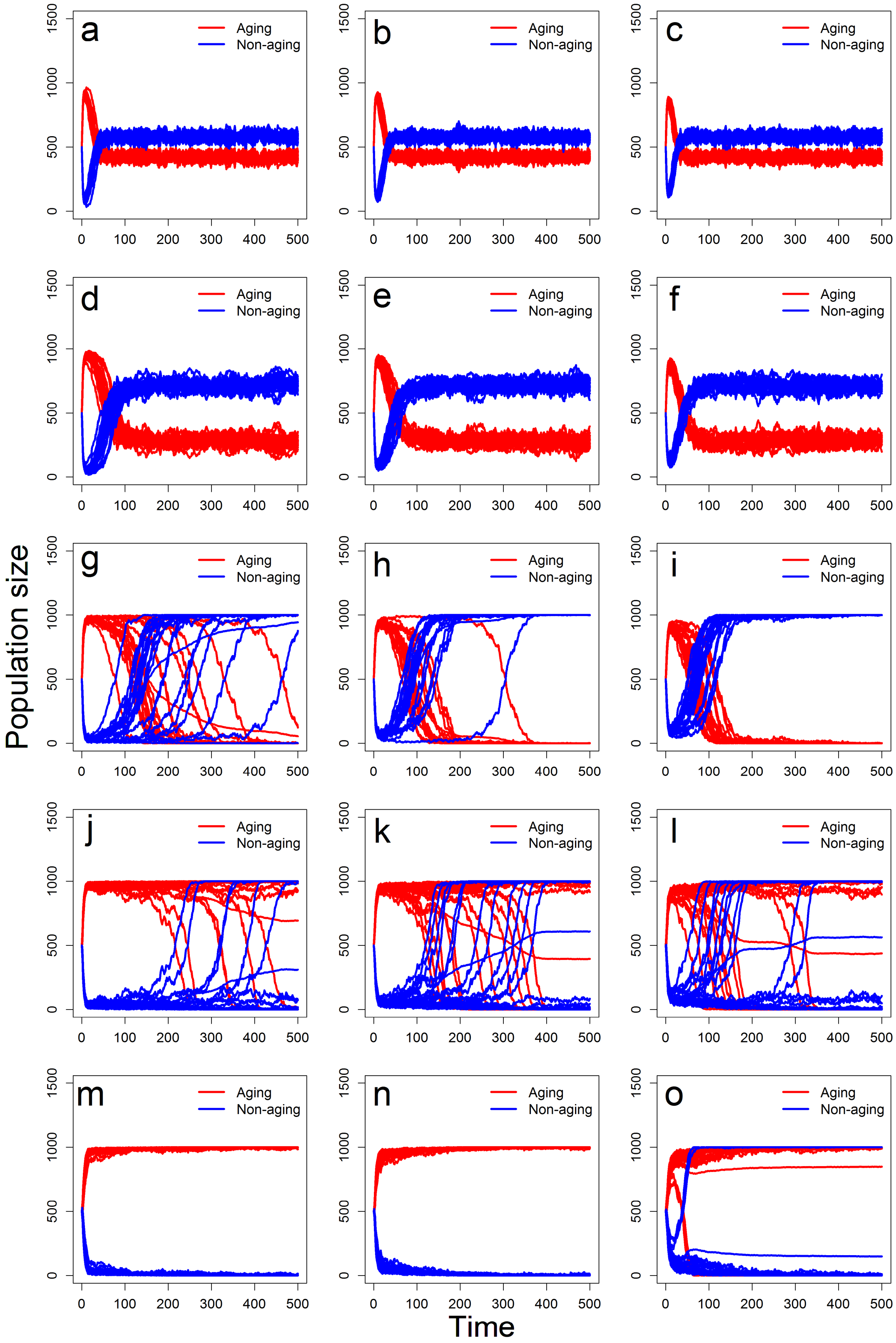

a
b
c
d
e
f
g
h
i
j
k
l
m
n
o
Aging
Non-aging
Population size
Time
0
500
1000
1500
0
100
200
300
400
500

Supplementary Figure S2: Representation of the aging and non-aging phenotypes in the population composed of simultaneous hermaphrodites under a variety of mating preferences and three different settings for the threshold rule (which determines the offspring phenotype and is quantified by the parameters p_f and p_g). Results are based on 20 simulation replicates and mating preferences are equal to (a-c) p_c = 0.3, (d-f) p_c = 0.4, (g-i) p_c = 0.5, (j-l) p_c = 0.6, and (m-o) p_c = 0.7. Moreover, the left column corresponds to parameters $p_f = 0.8$ and $p_g = 0.9$ (default values), the middle to $p_f = 0.7$ and $p_g = 0.8$ (less strict rule) and the right to $p_f = 0.6$ and $p_g = 0.7$ (even less strict rule). Other parameters are as in Table 1, with $b_0 = \beta = 1.5$, $p_s = 0.5$, and $P = 0$.

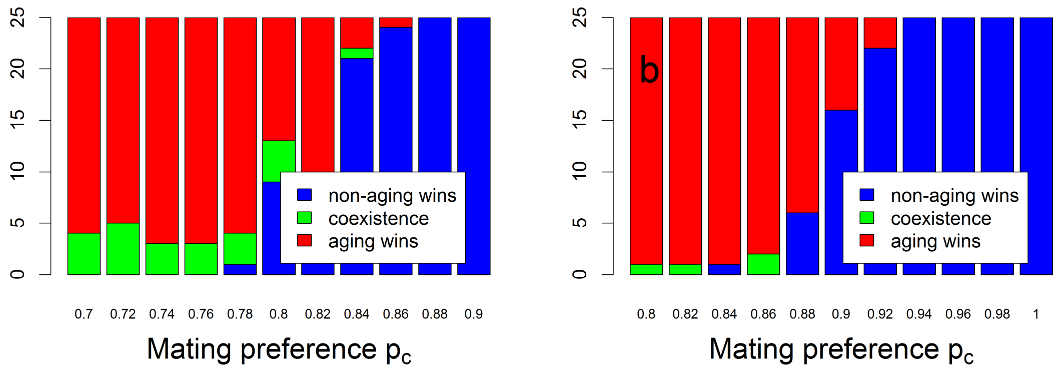

Supplementary Figure S3: The outcome of competition between aging and non-aging phenotypes in the two-sex population and the location of the p_c value at which the outcome tips from aging phenotype to non-aging phenotype domination. Other parameters are as in Table 1, with (a) $b_0 = \beta = 3$, (b) $b_0 = \beta = 6$, $p_s = 0.5$, and $P = 0$; based on 25 simulation replicates for each simulated scenario.

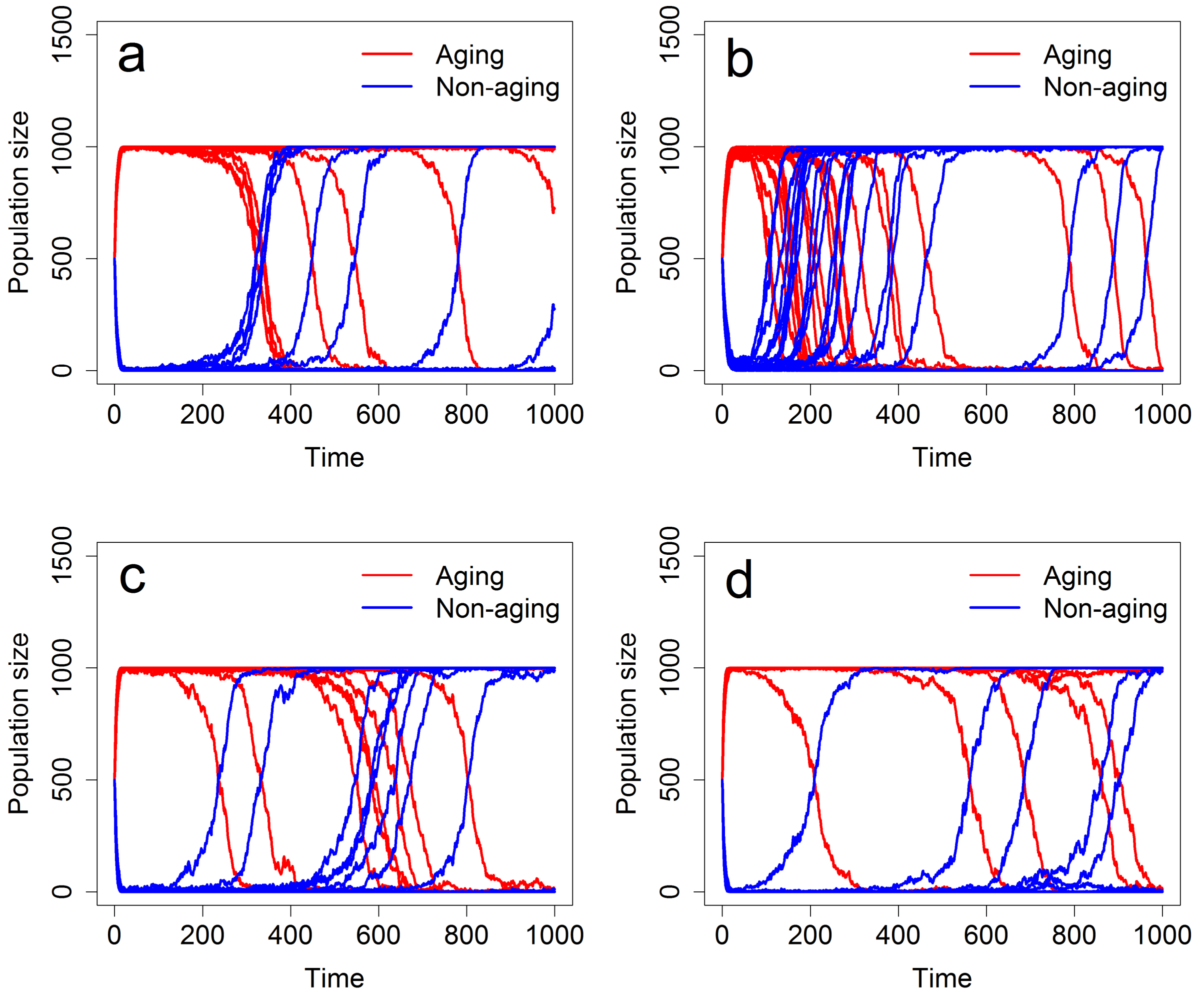

Supplementary Figure S4: Representation of the aging and non-aging phenotypes in the two-sex population under various parasite pressure. Results show a scenario without parasites (a), with parasites causing a fecundity reduction (b; $E_1 = 0.8, E_2 = 0$), and with parasites causing mortality enhancement (c; $E_1 = 0, E_2 = 0.2$, and d; $E_1 = 0, E_2 = 0.4$). Other parameters are as in Table 1, with $b_0 = \beta = 3, p_c = 0.5, p_s = 0.5$; 20 simulation replicates are run for each simulated scenario.
